# *Lactobacillus rhamnosus* GG Modulates Mitochondrial Function and Antioxidant Responses in an Ethanol-Exposed In Vivo Model: Evidence of HIGD2A-Dependent OXPHOS Remodeling in the Liver

**DOI:** 10.3390/antiox14060627

**Published:** 2025-05-23

**Authors:** Celia Salazar, Marlen Barreto, Alfredo Alfonso Adriasola-Carrasco, Francisca Carvajal, José Manuel Lerma-Cabrera, Lina María Ruiz

**Affiliations:** 1 Institute of Biomedical Sciences, Faculty of Health Sciences, Universidad Autónoma de Chile, Santiago 8580658, Chile; celia.salazar@uautonoma.cl (C.S.); marlen.barreto@uautonoma.cl (M.B.); alfredo.aac@gmail.com (A.A.A.-C.); 2 Department of Psychology, University of Almería, 04120 Almería, Spain; maria.carvajal@ual.es; 3 Health Research Center, CEINSA, University of Almeria, 04120 Almeria, Spain

**Keywords:** *Lactobacillus rhamnosus GG*, microbiota, mitochondrial physiology, binge-like ethanol exposure, NADH, ADP/ATP ratio, OXPHOS, *Higd2a*, *MnSOD*, *AMPKα1*

## Abstract

The gut microbiota plays a central role in host energy metabolism and the development of metabolic disorders, partly through its influence on mitochondrial function. Probiotic supplementation, particularly with *Lactobacillus rhamnosus* GG, has been proposed as a strategy to modulate the microbiota and improve host metabolic health. Adolescent binge-like alcohol consumption is a critical public health issue known to induce neuroinflammation, oxidative stress, mitochondrial dysfunction, and intestinal dysbiosis, contributing to disorders such as alcoholic liver disease (ALD). This study aimed to evaluate the effects of *L. rhamnosus* GG supplementation on mitochondrial physiology in Sprague Dawley rats exposed to binge-like ethanol (BEP group) or saline (SP group) during adolescence (postnatal days 30–43). Starting on postnatal day 44, *L. rhamnosus* GG was administered orally for 28 days. Fecal colonization was confirmed by qPCR, and mitochondrial function was assessed in the liver, heart, and bone marrow through quantification of NADH, ATP, ADP/ATP ratio, total antioxidant capacity, and the expression of mitochondrial genes *Higd2a*, *MnSOD1*, and *AMPKα1*. *L. rhamnosus* GG supplementation induced tissue-specific mitochondrial adaptations. In the liver, it increased *Higd2a* expression and restored antioxidant and energy balance in ethanol-exposed rats. In the bone marrow, it reversed ethanol-induced metabolic stress and enhanced *AMPKα1* expression. In contrast, in the heart, *L. rhamnosus* GG had minimal impact on mitochondrial energy markers but increased antioxidant capacity, indicating a more limited, redox-focused effect. These findings suggest that *L. rhamnosus* GG exerts context-dependent, tissue-specific benefits on mitochondrial physiology, primarily through the modulation of antioxidant defenses, activation of *AMPKα1*, and remodeling of respiratory complexes. This probiotic may represent a promising therapeutic strategy to mitigate mitochondrial dysfunction associated with early-life alcohol exposure.

## 1. Introduction

Adolescent alcohol consumption is a significant public health concern, characterized by the intake of large amounts of alcohol in a short period of time, known as binge drinking. This pattern of alcohol consumption has been linked to neuroinflammation, oxidative stress, and mitochondrial dysfunction, contributing to long-term neurological and metabolic impairments [1,2,3,4]. Recent studies have further highlighted that binge drinking during adolescence leads to persistent mitochondrial abnormalities in the brain, particularly affecting oxidative phosphorylation, adenosine triphosphate (ATP) production, and reactive oxygen species (ROS) homeostasis [5,6].

The gut microbiota plays a critical role in host metabolism, immune function, and neurodevelopment, comprising approximately 70 distinct bacterial genera that form the intestinal microbiome [7]. However, microbial composition and activity are influenced by environmental factors, including diet, alcohol consumption, and probiotic intake. The metabolic state of these bacterial populations can fluctuate between active and latent states, underscoring the importance of understanding gut microbiota function in various physiological contexts [8,9,10].

Probiotic supplementation has emerged as a promising strategy to modulate the composition and function of the gut microbiota, thereby influencing host health [11]. Certain bacterial strains, including *Lactobacillus rhamnosus* GG (*L. rhamnosus* GG), have been shown to have protective effects against metabolic disorders, gut dysbiosis, and alcohol-induced oxidative stress [12,13]. In particular, probiotic administration has been associated with the modulation of inflammatory pathways, improved mitochondrial function, and enhanced gut barrier integrity [14,15]. Despite evidence supporting the beneficial effects of probiotics in obesity, metabolic syndrome, and alcohol-related gut dysbiosis, little is known about how *L. rhamnosus* GG explicitly modulates the dynamics of active gut microbiota and mitochondrial physiology [13,14].

Studies indicate that *L. rhamnosus* GG alters gut microbiota composition, increasing species diversity and promoting beneficial genera such as *Lactobacillus* and *Bifidobacterium* [16,17]. Furthermore, *L. rhamnosus* GG has been shown to increase total bacterial load in mesenteric lymph nodes, which may enhance mucosal immunity and metabolic resilience in response to dietary and environmental stressors [16,18].

Beyond gut microbiota interactions, mitochondria share evolutionary and functional similarities with bacteria due to their endosymbiotic origin [19]. This shared ancestry allows direct communication between microbiota-derived metabolites and mitochondrial biogenesis pathways [20,21]. Specifically, short-chain fatty acids (SCFAs), such as propionate, influence mitochondrial metabolism by modulating tricarboxylic acid (TCA) cycle activity, ATP production, and responses to oxidative stress [22,23,24]. Recent studies have emphasized that gut microbiota composition, particularly the relative abundance of *Bacteroidetes*, influences SCFA production, which can disrupt mitochondrial homeostasis [25,26].

Mitochondrial dysfunction is increasingly recognized as a key factor in neurological and metabolic disorders linked to gut dysbiosis [27,28,29]. In particular, Bacteroidetes-dominant microbiota profiles have been implicated in excessive propionate production, which interferes with mitochondrial respiration and ATP generation [30,31,32]. Given that mitochondrial function is crucial for maintaining the gut–brain axis, alterations in microbiota composition may have profound consequences on neurological health, metabolic regulation, and immune function [20,21].

Therefore, understanding how probiotic supplementation affects mitochondrial function in response to environmental stressors, such as binge drinking, is critical. Given that microbiota quality and diversity modulate mitochondrial efficiency and host metabolism, the present study aimed to investigate whether dietary supplementation with *L. rhamnosus* GG could counteract the mitochondrial dysfunction and oxidative stress induced by binge-like ethanol exposure during adolescence. We specifically sought to assess changes in mitochondrial bioenergetics parameters, oxidative stress markers, and gut–mitochondrial composition in response to probiotic treatment. It is hypothesized that supplementation with *L. rhamnosus* GG would mitigate ethanol-induced mitochondrial dysfunction by preserving oxidative phosphorylation efficiency, reducing oxidative stress and modulating gut microbiota composition in a manner that supports mitochondrial resilience.

## 2. Materials and Methods

### 2.1. Study Design and Animals

**Figure 1:**
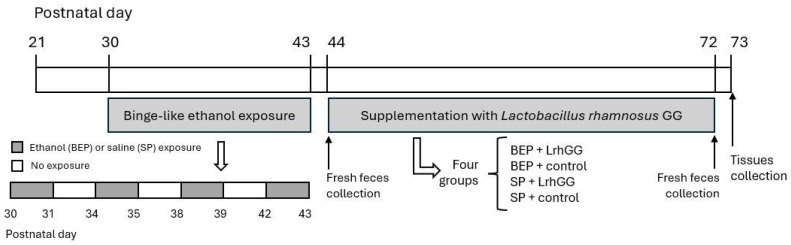
Schematic of the experimental design. Abbreviations: BEP, ethanol exposure; LrhGG, *Lactobacillus rhamnosus GG*; SP, saline exposure.

Sprague Dawley rats from the Bioterium of the Center for Medical Research (CIM) at the Pontificia Universidad Católica de Chile were housed individually in an environmentally controlled room (22 °C temperature, 12:12 h light-dark cycle) in the bioterium of the Institute of Biomedical Sciences at the Universidad Autónoma de Chile, with standard rodent chow and water provided ad libitum. All animal procedures were conducted in strict accordance with ethical guidelines for the care and use of laboratory animals. The study was approved by the Scientific Ethics Committee of the Universidad Autónoma de Chile (Act No. 22-06-2015). All procedures complied with the requirements of the Chilean National Commission for Scientific and Technological Research (CONICYT) (https://www.conicyt.cl/fondecyt/files/2012/10/Libro-4-Aspectos-Bio%C3%A9ticos-de-la-Experimentaci%C3%B3n-Animal.pdf, accessed on 19 May 2025 [33]) and were conducted in accordance with the guidelines outlined in the NIH Guide for the Care and Use of Laboratory Animals (1996). Additionally, animal handling adhered to the principles outlined in the U.K. Animals (Scientific Procedures) Act 1986 and the European Union Directive 2010/63/EU on the protection of animals used for scientific purposes. The study employed a binge-like ethanol exposure model (Figure 1 and Table 1), simulating this pattern of alcohol intake during adolescence in humans (from postnatal day (PND) 30 to PND 43).

Animals were divided into adolescent binge-like ethanol exposure (BEP) or saline (SP) groups. Morning doses of either 25% (*w*/*v*) ethanol (2.5 g/kg) in isotonic saline (BEP) or saline (SP) were administered intraperitoneally (i.p.) to 30-day-old pups on two consecutive days, with a 2-day gap between injections, for 2 weeks (Figure 1). Specifically, pups were injected on postnatal days (PNDs) 30, 31, 34, 35, 38, 39, 42, and 43 [34,35].

Rats in both conditions were divided into two groups: *L. rhamnosus* GG (LrhGG) (BEP-LrhGG or SP-LrhGG) and a control group receiving plain drinking water (BEP-control or SP-control). *L. rhamnosus* GG was administered via drinking water at a dose of 10^10^ colony-forming units (CFU) per day. Animals were treated with *L. rhamnosus* GG from PND 44 to PND 72. Animals in the plain drinking water group were treated similarly but did not receive *L. rhamnosus* GG. The dose of 10^10^ CFU of *L. rhamnosus* GG was selected based on previous studies demonstrating its effectiveness in enabling fecal detection of the microorganism [36,37]. This dosage has also been shown to elicit physiological effects, including anti-inflammatory, antioxidant, and hepatoprotective responses in vivo [38]. Furthermore, research in neonatal foals indicated that 10^10^ CFU was the most effective dose for enhancing immune function and antioxidant activity [39]. Importantly, this dosage has been validated as safe and non-toxic in rat models [40].

Fecal samples were collected before the start of *L. rhamnosus* GG supplementation (PND 43) and on the last day of supplementation (PND 72). Three fecal samples were collected from each animal and stored in Eppendorf tubes at −80 °C until further analysis. Animals were sacrificed between PND 73 and 74, and organs were harvested and stored at −80 °C until further analysis.

### 2.2. Tissue Selection and Collection

Liver, heart, and bone marrow tissues were selected for mitochondrial analysis based on their distinct metabolic roles and known susceptibility to ethanol-induced mitochondrial dysfunction. The liver is the central organ for ethanol metabolism via alcohol dehydrogenase and CYP2E1 pathways, leading to high ROS production and mitochondrial remodeling [41]. The heart, due to its high mitochondrial density and continuous energy demand, is particularly vulnerable to disruptions in oxidative phosphorylation and ATP production under ethanol stress [42]. The bone marrow, a redox-sensitive tissue critical for hematopoiesis, is increasingly recognized as a site of gut microbiota modulation and systemic oxidative stress response [43,44].

At the end of the treatment period, animals were euthanized under deep anesthesia. The liver and heart were quickly excised, rinsed in cold phosphate-buffered saline (PBS), blotted dry, and processed immediately for mitochondrial isolation or snap-frozen in liquid nitrogen for RNA and protein extraction. Bone marrow was collected by flushing femurs and tibias with cold PBS using a 27G needle. The cell suspension was filtered through a 70 μm cell strainer and centrifuged at 800× *g* for 10 min at 4 °C. Pellets were either processed for mitochondrial isolation or stored at −80 °C for molecular analysis.

### 2.3. Bacterial Strains and Culture Conditions

*L. rhamnosus* GG was cultured in de Man, Rogosa, and Sharpe (MRS) broth (Difco Laboratories Inc., Franklin Lakes, NJ, USA) under standard aerobic conditions at 37 °C. Bacterial cultures were incubated for 8 h to reach the late logarithmic growth phase, ensuring optimal viability for administration. After incubation, the cultures were harvested by centrifugation at 16,000× *g* for 5 min at 4 °C and washed twice with sterile phosphate-buffered saline (PBS) to remove residual media components. The bacterial pellet was resuspended in PBS and adjusted to a final concentration of 1 × 10^7^ CFU/mL. Bacterial concentration was standardized using a pre-established optical density (OD) calibration curve at 600 nm, which correlated OD measurements with colony-forming units (CFU). Optical density was measured with a SPECTROstar Nano spectrophotometer (BMG Labtech, Durham, NC, USA). For the intervention, 200 μL of the prepared *L. rhamnosus* GG suspension was administered via the drinking water throughout the treatment period (from PND 44 to PND 72), following established protocols [13].

### 2.4. Quantification of L. rhamnosus GG by Quantitative Polymerase Chain Reaction (qPCR)

Feces stored at −80 °C were processed to obtain the bacterial pellet following the protocol described by Ahlroos and Tynkkynen (2009) [36]. gDNA from *L. rhamnosus* GG was isolated using a NucleoSpin kit (Thermo Fisher Scientific, Waltham, MA, USA) according to the manufacturer’s protocol. The concentration of *L. rhamnosus* GG was determined using quantitative polymerase chain reaction (qPCR). The sample Ct was compared with that of the standard curve constructed from pure cultures of *L. rhamnosus* GG or feces inoculated with known concentrations of *L. rhamnosus* GG as described by Ahlroos and Tynkkynen (2009) [36]. The qPCR was performed using the FastStart Essential DNA Green Master Kit (Roche, Basel, Switzerland) on a LightCycler^®^ 96 Real-Time PCR System (Roche). The primer sequences used were (F) 5′-CGCCCTTAACAGCAGTCTTC-3′ and (R) 5′-GCCCTCCGTATGCTTAAACC-3′ [36]. The amplification program consisted of 45 repeated cycles, which included denaturation for 2 s at 95 °C, annealing for 15 s at 63 °C, and chain extension for 32 s at 72 °C [36]. The specificity of amplification was confirmed by melting curve analysis.

### 2.5. Reverse Transcriptase and Quantitative Real-Time PCR (qRT-PCR)

Total RNA was extracted from rat bone marrow, liver, and heart tissues using TRIzol reagent (Invitrogen Carlsbad, CA, USA) following the manufacturer’s protocol. RNA concentration and purity were assessed using an Infinite M200 Pro spectrophotometer (TECAN, Männedorf, Switzerland), and RNA integrity was evaluated by agarose gel electrophoresis. For cDNA synthesis, two µg of total RNA was reverse-transcribed using the RevertAid First Strand cDNA Synthesis Kit (Thermo Fisher Scientific, Waltham, MA, USA) according to the manufacturer’s instructions. Quantitative PCR (qPCR) was performed using the FastStart Essential DNA Green Master Kit (Roche) on a LightCycler^®^ 96 Real-Time PCR System (Roche). Gene expression was analyzed for HIG1 Hypoxia Inducible Domain Family Member 2A (*Higd2a)*, and results were normalized to the reference gene *PPIA*. An additional housekeeping gene, glyceraldehyde-3-phosphate dehydrogenase (GAPDH), was also included for validation. The primer sequences used were: *Higd2a* Fw: 5′-GCCTTTTGATCCGTCCAAGC-3′, Rev: 5′-CTGAAACGGAGGGAGCAAGT-3′; *PPIA* Fw: 5′-GTGGTCTTTGGGAAGGTG-3′, Rev: 5′-GGTGATCTTCTTGCTGGTC-3′; *GAPDH* Fw: 5′-ACCACAGTCCATGCCATCAC-3′, Rev: 5′-TCCACCACCCTGTTGCTGTA-3′. Thermal cycling conditions were as follows: an initial three-step amplification (95 °C for 10 s, 60 °C for 10 s, and 72 °C for 10 s), followed by a one-step melting (95 °C for 10 s, 65 °C for 60 s, and 97 °C for 1 s) and finishing with a one-step cooling (37 °C for 30 s). All qPCR reactions were completed with a melting curve analysis to verify the specificity of the amplification.

### 2.6. Mitochondrial Isolation

Mitochondria were isolated using the standard differential centrifugation method described by Schnaitman and Greenawalt (1968) [45]. Tissues were minced in 10 volumes of cold mitochondrial isolation buffer, which contained 70 mM sucrose, 210 mM mannitol, 5 mM HEPES, 1 mM EGTA, and 0.5% (*w*/*v*) fatty acid-free bovine serum albumin (pH 7.2). All steps were carried out at 4 °C, and samples were kept on ice throughout the procedure. Tissue samples were rinsed several times with cold buffer to remove residual culture medium. Mechanical disruption was performed using a Dounce homogenizer with 20 strokes using the loose pestle, followed by 20 strokes with the tight pestle to ensure thorough cell lysis. The homogenate was first centrifuged at 800× *g* for 10 min at 4 °C to remove cell debris and nuclei. The resulting supernatant was then centrifuged at 8000× *g* for 10 min at 4 °C to pellet the mitochondria. The final mitochondrial pellet was resuspended in 100 μL of ice-cold mitochondrial isolation buffer and stored on ice for immediate downstream applications.

### 2.7. Western Blotting (WB)

Mitochondrial proteins were extracted from freshly isolated mitochondria and solubilized in a lysis buffer containing 20 mM HEPES, 2 mM EDTA, 0.5% Triton X-100, 150 mM NaCl, 1 mM PMSF, and a 1× HALT™ protease inhibitor cocktail (Thermo Fisher Scientific, Waltham, MA, USA). Samples were incubated on ice for 30 min to ensure complete solubilization.

Proteins were separated by 12% SDS-PAGE using a Tris-Glycine running buffer system and subsequently transferred to a 0.2 μm PVDF membrane via the wet transfer method.

Protein detection was performed using the following primary antibodies: anti-HIGD2A antibody (Mouse anti-Higd2a ab135399 Abcam, Cambridge, UK); anti-VDAC1 antibody (ab15895 Abcam); anti-β-Actin antibody (ab75186 Abcam); total oxidative phosphorylation (OXPHOS) rodent WB antibody cocktail (ab110413 Abcam). Detection was performed using Pierce ECL Western Blotting Substrate (Thermo Fisher Scientific), and chemiluminescent signals were captured using a suitable imaging system. Band intensities were quantified using ImageJ software version 1.54p. Protein levels were normalized to β-actin as an internal control for loading.

### 2.8. Total Antioxidant Capacity

The total antioxidant capacity was assessed using the OxiSelect Total Antioxidant Capacity (TAC) Assay Kit (Cat. # STA-360, Cell Biolabs, Inc., San Diego, CA, USA), following the manufacturer’s instructions. This assay measures the ability of antioxidants in the sample to reduce copper (II) to copper (I), which then reacts with a chromogenic reagent to produce a colorimetric signal [46]. Results are expressed as micromolar (μM) copper-reducing equivalents (CRE), as indicated by the standard curve provided with the kit.

### 2.9. ADP/ATP Ratio Assay

The ADP/ATP Ratio Assay Kit (Bioluminescent, Cat. # ab65313, Abcam) was used to measure ADP and ATP levels in mitochondria isolated from bone marrow, liver, and heart tissues. The assay was performed according to the manufacturer’s protocol, which involves the enzymatic conversion of ADP to ATP, followed by detection using a luciferase-based bioluminescence system. Luminescence was measured using an Infinite M200 Pro microplate reader (TECAN, Männedorf, Switzerland), and the ADP/ATP ratio was calculated based on the luminescent signal intensities.

### 2.10. NADH Levels Were Quantified by Autofluorescence Analysis

NADH levels were quantified in mitochondrial protein extracts using a fluorescence-based autofluorescence method [47]. Mitochondrial fractions were prepared as described previously, and protein extracts were loaded into Costar^®^ 96-well black flat-bottom plates (Costar^®^ Arlington, Polystyrene, Arlington, VA, USA,; Cat. No. 3991, 3650, 3916, 3915, 3925). Fluorescence was measured using an Infinite^®^ 200 Pro microplate reader (TECAN) equipped with i-Control software version 1.10.4.0, in top-read fluorescence mode. The following settings were applied: excitation wavelength, 324 nm; emission wavelength, 461 nm; excitation bandwidth, 9 nm; emission bandwidth, 20 nm; gain, 100; number of flashes, 25; integration time, 100 μs; temperature, 25.1 °C. Fluorescence intensity was used as an indirect measure of NADH concentration, as NADH exhibits intrinsic fluorescence under the specified conditions.

### 2.11. Statistical Analysis

All statistical analyses were performed using GraphPad Prism version 6 (GraphPad Software, San Diego, CA, USA). For comparisons between two groups, an unpaired Student’s *t*-test was applied. When comparing more than two groups, a one-way ANOVA was conducted, followed by Tukey’s or Dunnett’s post hoc multiple comparison tests, as appropriate. A *p*-value of <0.05 was considered statistically significant. All data are presented as mean ± standard error of the mean (SEM) unless otherwise stated.

## 3. Results

### 3.1. Lactobacillus rhamnosus GG Modulates Mitochondrial Gene Expression and Antioxidant Defense in Response to Binge-like Ethanol Exposure

The administration of *L. rhamnosus* GG led to differential gene expression in various tissues of rats exposed to ethanol (BEP) or saline (SP) during adolescence, suggesting potential effects on mitochondrial function. *L. rhamnosus* GG was detected in the feces of treated rats at levels of 10^5^–10^7^ CFU, while it remained undetectable in the control group (Supplementary Figure S1). Previous behavioral assessments using the elevated plus maze (EPM) in adult Sprague Dawley rats showed that BEP animals exhibited increased anxiety-like behaviors, including stretch-attend posture, exploration, and grooming, compared to SP controls. However, no significant differences were observed in classical EPM parameters. Notably, although dietary administration of LrhGG increased its presence in feces in both groups, it did not alter anxiety-related behavior [48].

**Figure 2:**
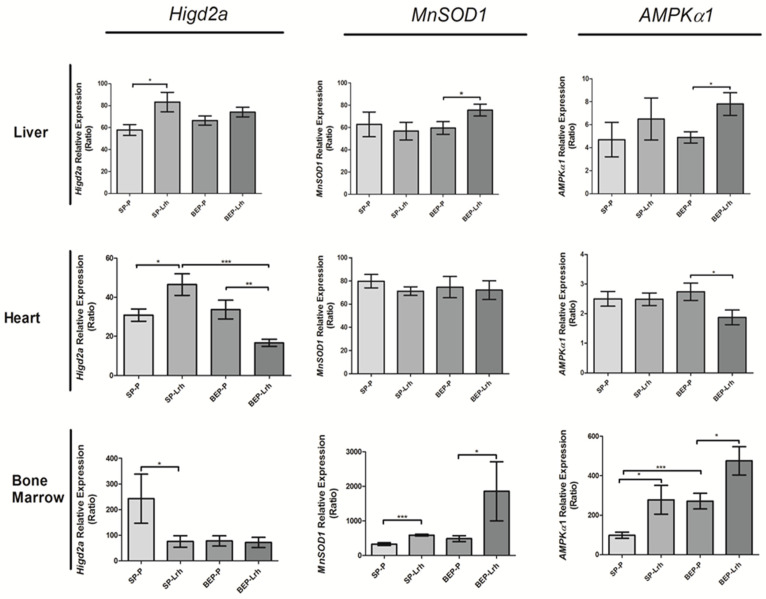
Relative gene expression of *Higd2a*, *MnSOD1*, and *AMPKα1* in the liver, heart, and bone marrow of rats exposed to ethanol (BEP) or saline (SP) during adolescence, following treatment with *Lactobacillus rhamnosus* GG (Lrh) or water (P). Data are presented as mean ± SEM (*n* = five biological replicates per group, with two technical replicates per sample). Statistical analysis was performed using one-way ANOVA, followed by Dunnett’s post hoc test, with significance set at *p* < 0.05. * *p* < 0.05; ** *p* < 0.01; *** *p* < 0.001.

To analyze mitochondrial function, we used qPCR to examine the expression of the Higd2a gene, which encodes the mitochondrial protein Higd2a, a mediator of respirosome assembly—a supramolecular complex comprising respiratory complexes III and IV. We analyzed the gene expression of the antioxidant enzyme Manganese Superoxide Dismutase (*MnSOD1*) and AMP-activated protein kinase (*AMPKα1*), a central sensor of cellular energy balance. In the liver, *L. rhamnosus* GG significantly increased the expression of *Higd2a* in the SP group while also inducing *MnSOD1* and *AMPKα1* expression in both the SP and BEP groups, indicating enhanced antioxidant defense and energy regulation (Figure 2). In the heart, *L. rhamnosus* GG significantly upregulated *Higd2a* expression in the SP group.

In contrast, binge-like ethanol exposure reduced both *Higd2a* and *AMPKα1*, suggesting tissue-specific modulation of mitochondrial activity in response to ethanol exposure (Figure 2). Notably, in the bone marrow, *L. rhamnosus* GG treatment reduced Higd2a expression in both the SP and BEP groups, while increasing *MnSOD1* and *AMPKα1*, thereby reinforcing its role in oxidative stress regulation (Figure 2). These findings suggest that *L. rhamnosus* GG exerts its beneficial effects by remodeling respiratory complexes, enhancing antioxidant defenses, and regulating cellular energy balance. The observed tissue-specific responses, particularly the suppression of *Higd2a* and *AMPKα1* in the heart of the BEP group, highlight the potential adaptive mechanisms of mitochondrial physiology following early-life ethanol exposure.

### 3.2. Lactobacillus rhamnosus GG Modulates Mitochondrial Physiology in Rats Exposed to Binge-like Ethanol During Adolescence

**Figure 3:**
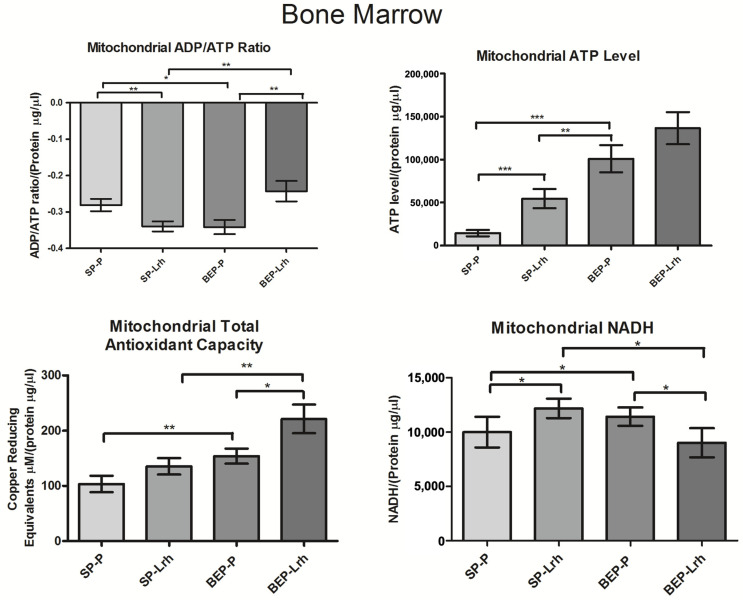
NADH levels, ADP/ATP ratio, and total antioxidant capacity in the bone marrow of rats exposed to ethanol (BEP) or saline (SP) during adolescence following *Lactobacillus rhamnosus GG* (Lrh) or water (P) treatment. Data are presented as mean ± SEM (*n* = five biological replicates per group, with two technical replicates per sample). Statistical analysis was performed using one-way ANOVA, followed by Dunnett’s post hoc test, with significance set at *p* < 0.05. * *p* < 0.05; ** *p* < 0.01; *** *p* < 0.001.

**Figure 4:**
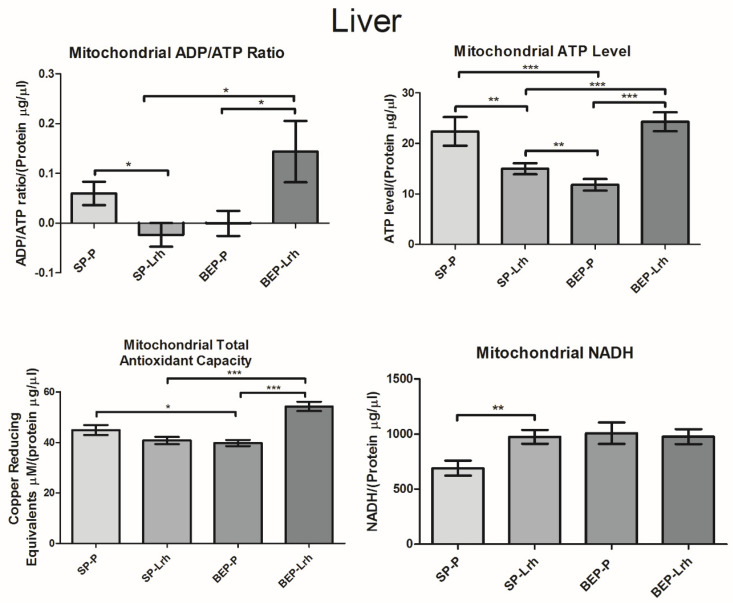
NADH levels, ADP/ATP ratio, and total antioxidant capacity in the liver of rats exposed to ethanol (BEP) or saline (SP) during adolescence following *Lactobacillus rhamnosus GG* (Lrh) or water (P) treatment. Data are presented as mean ± SEM (*n* = five biological replicates per group, with two technical replicates per sample). Statistical analysis was performed using one-way ANOVA, followed by Dunnett’s post hoc test, with significance set at *p* < 0.05. * *p* < 0.05; ** *p* < 0.01; *** *p* < 0.001.

**Figure 5:**
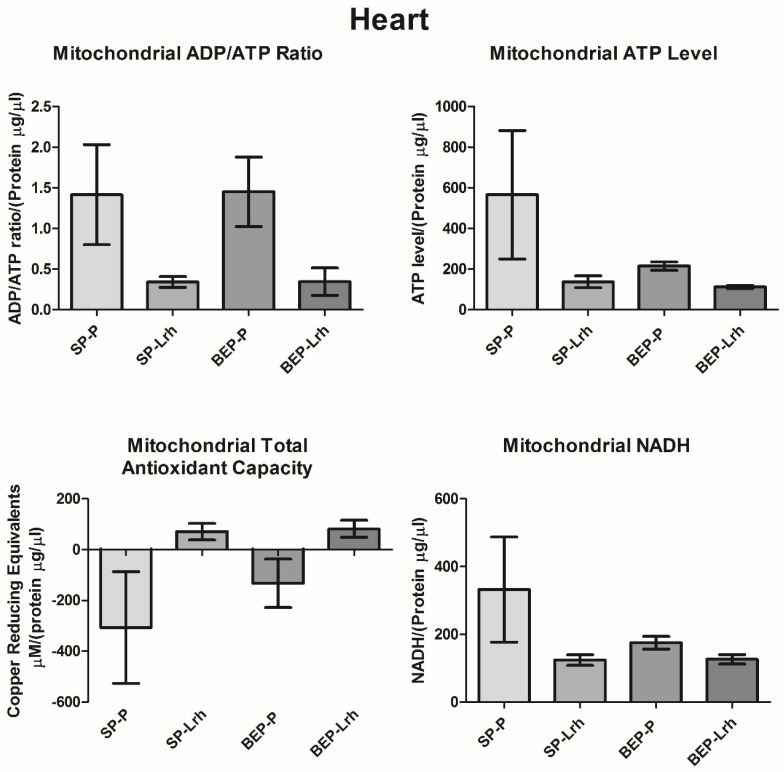
NADH levels, ADP/ATP ratio, and total antioxidant capacity in the heart of rats exposed to ethanol (BEP) or saline (SP) during adolescence following *Lactobacillus rhamnosus* GG (Lrh) or water (P) treatment. Data are presented as mean ± SEM (*n* = five biological replicates per group, with two technical replicates per sample). Statistical analysis was performed using one-way ANOVA, followed by Dunnett’s post hoc test, with significance set at *p* < 0.05.

Mitochondrial NADH, an indicator of cellular respiration, exhibited a significant increase in the SP group supplemented with *L. rhamnosus* GG and the BEP group. In contrast, NADH levels in the BEP group supplemented with *L. rhamnosus* GG were regularized (Figure 3). In the liver, NADH levels increased significantly in both SP and BEP groups following *L. rhamnosus* GG treatment (Figure 4). Similarly, *L. rhamnosus* GG enhanced mitochondrial total antioxidant capacity and ATP levels in the bone marrow of both groups, with a significant increase observed in the liver of the BEP group (Figure 3). The ADP/ATP ratio, a key indicator of cellular energy balance, was significantly reduced in the bone marrow and liver of the BEP group without *L. rhamnosus* GG and in the SP group treated with *L. rhamnosus* GG, suggesting increased cellular proliferation. However, in the BEP group treated with *L. rhamnosus* GG, the ADP/ATP ratio was restored to normal levels (Figure 3 and Figure 4). In contrast, in the heart, NADH levels, ATP levels, and the ADP/ATP ratio decreased significantly in the SP and BEP groups treated with *L. rhamnosus* GG. In contrast, the mitochondrial total antioxidant capacity increased (Figure 5). These findings suggest that *L. rhamnosus* GG modulates mitochondrial function through enhanced respiratory activity, regulation of oxidative stress, and modulation of energy metabolism, with tissue-specific responses influenced by prior exposure to ethanol.

### 3.3. HIGD2A Expression Correlates with OXPHOS Complex Remodeling in Ethanol-Exposed Rats

**Figure 6:**
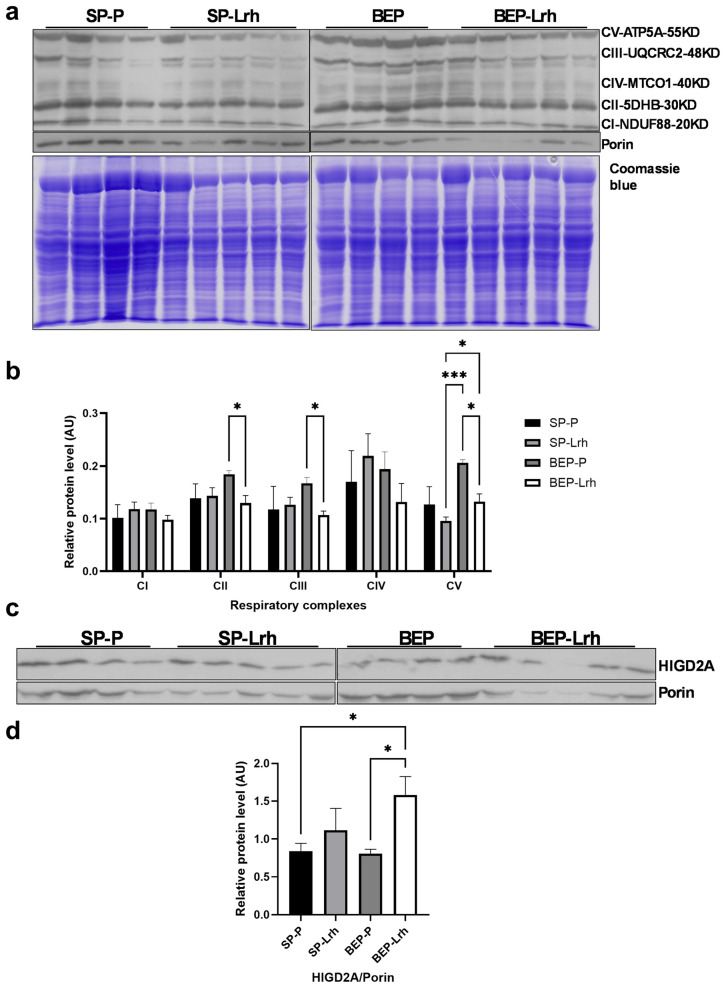
Changes in *Higd2a* expression are associated with remodeling of the OXPHOS complex. (**a**) Western blot analysis of OXPHOS complexes using the Total OXPHOS Rodent WB Antibody Cocktail (ab110413). (**b**) Quantification of OXPHOS complexes normalized to total protein loading, as determined by SDS-PAGE Coomassie blue staining. Data are presented as mean ± SEM (*n* = five biological replicates). Statistical analysis was performed using Two-way ANOVA (or Mixed Model), followed by mixed-effects analysis multiple comparisons, with a significance level set at *p* < 0.05. (**c**) Western blot analysis of Higd2a protein in liver mitochondrial extracts from rats exposed to ethanol (BEP) or saline (SP) during adolescence and treated with *Lactobacillus rhamnosus* GG (Lrh) or water (P). (**d**) Quantification of Higd2a protein levels normalized to Porin. Data are presented as mean ± SEM (*n* = five biological replicates). Statistical analysis was performed using one-way ANOVA, followed by Fisher’s LSD test, with a significance level set at *p* < 0.05. * *p* < 0.05; *** *p* < 0.001.

The expression of HIGD2A is closely linked to the remodeling of oxidative phosphorylation (OXPHOS) complexes in this in vivo model. In the liver of ethanol-exposed rats treated with *L. rhamnosus* GG (BEP-Lrh group), HIGD2A protein expression was significantly increased, while the expression of all five OXPHOS complexes (CI–CV) was concurrently reduced (Figure 6).

This inverse relationship suggests that *L. rhamnosus* GG supplementation restores mitochondrial remodeling capacity under ethanol-induced stress conditions, potentially by promoting the assembly or stabilization of respiratory supercomplexes through HIGD2A upregulation. These findings reinforce the proposed regulatory role of HIGD2A in mitochondrial structural and functional adaptation, particularly in response to dietary interventions and environmental stressors such as binge-like alcohol exposure.

## 4. Discussion

The results of this study demonstrate that *L. rhamnosus* GG modulates mitochondrial function in a tissue-dependent manner, influencing cellular respiration, oxidative stress regulation, and energy balance. The differential gene expression patterns observed across tissues suggest that *L. rhamnosus* GG promotes mitochondrial efficiency under normal conditions (SP group) and restores mitochondrial homeostasis following ethanol exposure (BEP group) (Figure 2). These findings offer valuable insights into how probiotics affect mitochondrial dynamics and the remodeling of oxidative phosphorylation (OXPHOS).

*L. rhamnosus* GG supplementation significantly increased the expression of *Higd2a*, *MnSOD1*, and *AMPKα1* in the liver of SP and BEP groups, supporting its role in enhancing mitochondrial biogenesis, antioxidant defense, and energy regulation (Figure 2). The increased *Higd2a* expression in the SP group suggests that *L. rhamnosus* GG optimizes mitochondrial respiratory efficiency under normal conditions. *Higd2a* encodes Higd2a, a protein that facilitates the assembly of respiratory supercomplexes, particularly complexes III and IV, which are crucial for optimal electron transport chain (ETC) function [49,50]. This aligns with studies reporting that probiotics enhance mitochondrial bioenergetics by stabilizing OXPHOS supercomplexes and reducing electron leakage [51].

Ethanol disrupts mitochondrial protein homeostasis and increases oxidative damage, leading to adaptive upregulation of ETC components to maintain ATP production [6]. These results indicate that Higd2a plays a regulatory role in mitochondrial adaptation, as its expression correlates with oxidative phosphorylation (OXPHOS) remodeling in response to metabolic stress.

*L. rhamnosus* GG enhanced *MnSOD1* and *AMPKα1* expression in the bone marrow of both SP and BEP groups, reinforcing its role in regulating oxidative stress and maintaining metabolic homeostasis (Figure 1). Increased *MnSOD1* expression suggests that *L. rhamnosus* GG strengthens mitochondrial antioxidant defenses, reducing reactive oxygen species (ROS) levels and preventing oxidative damage [52,53]. The concurrent upregulation of *AMPKα1* suggests improved cellular energy sensing and ATP production, consistent with findings that probiotics activate AMPK pathways to optimize mitochondrial quality control [54,55].

Interestingly, *Higd2a* expression decreased in the bone marrow of both SP and BEP groups despite enhanced antioxidant and energy regulatory responses (Figure 2). This suggests a distinct mitochondrial remodeling strategy in hematopoietic cells, prioritizing the control of oxidative stress over the formation of respiratory supercomplexes. Previous studies have indicated that probiotic supplementation can modulate hematopoietic stem cell metabolism by regulating mitochondrial reactive oxygen species (ROS) levels and ATP availability, which are essential for sustaining cellular proliferation and differentiation [44,56].

Additionally, the ADP/ATP ratio significantly decreased in the SP group supplemented with *L. rhamnosus* GG and in the BEP group without *L. rhamnosus* GG, indicating enhanced ATP production and metabolic activity. However, in the BEP group treated with *L. rhamnosus* GG, the ADP/ATP ratio returned to normal levels, indicating that *L. rhamnosus* GG restores mitochondrial energy balance under ethanol-exposed conditions (Figure 2). This homeostatic effect supports the hypothesis that *L. rhamnosus* GG optimizes ATP utilization and mitochondrial efficiency, preventing excessive metabolic shifts that could lead to mitochondrial dysfunction.

Unlike the liver and bone marrow, the heart exhibited a distinct metabolic response to *L. rhamnosus* GG supplementation. NADH levels, ATP levels, and the ADP/ATP ratio decreased significantly in both the SP and BEP groups, while total mitochondrial antioxidant capacity increased (Figure 5). Chronic ethanol exposure impairs mitochondrial respiration and AMPK signaling in cardiomyocytes, leading to metabolic stress and ATP depletion [57]. The observed decrease in NADH and ATP suggests a metabolic shift towards a more energy-efficient state, potentially as a protective mechanism against mitochondrial overload.

The downregulation of *Higd2a* and *AMPKα1* in the heart of the BEP group treated with *L. rhamnosus* GG may indicate a shift toward an alternative metabolic state to compensate for ethanol-induced mitochondrial damage (Figure 2). Similar findings have been reported in models of ethanol-induced mitochondrial dysfunction, where cells reduce mitochondrial activation to prevent excessive ROS production and protect cardiac tissue [6]. Despite reduced mitochondrial activity, the increase in antioxidant capacity suggests that *L. rhamnosus* GG still exerts a protective effect by reinforcing cellular defenses against oxidative stress.

The observed differential expression of Higd2a and its association with OXPHOS complex remodeling (Figure 5) suggest that this protein plays a central role in mitochondrial bioenergetics. In the SP group, Higd2a upregulation coincided with downregulation of all OXPHOS complexes (Figure 5), suggesting that *L. rhamnosus* GG enhances mitochondrial efficiency by promoting supercomplex stability, reducing the need for high individual ETC component expression [58]. Conversely, the opposite trend in the BEP group—Higd2a downregulation with increased OXPHOS complex expression (Figure 5)—suggests a compensatory response to mitochondrial stress, requiring higher ETC activity to maintain energy production.

These findings align with research showing that Higd2a promotes respiratory super complex formation, which optimizes ATP synthesis and minimizes electron leakage [50,59]. The results suggest that *L. rhamnosus* GG-induced changes in Higd2a expression may drive tissue-specific metabolic adaptations, depending on prior exposure to ethanol. Further studies are needed to determine whether targeting Higd2a could be a viable strategy to optimize mitochondrial function in metabolic disorders associated with oxidative stress.

This study demonstrates that *L. rhamnosus* GG exerts tissue-dependent effects on mitochondrial function, influencing energy metabolism, oxidative stress regulation, and OXPHOS remodeling. *L. rhamnosus* GG enhanced antioxidant defenses and ATP production in the liver and bone marrow. In the heart, it induced a protective metabolic shift in response to ethanol exposure. The findings reinforce the role of Higd2a as a key regulator of mitochondrial adaptation, with its expression modulating the activity of the OXPHOS complex in response to metabolic demands.

In the heart, mitochondrial analysis revealed that both SP and BEP rats treated with *L. rhamnosus* GG exhibited a significant reduction in NADH and ATP levels (Figure 5), suggesting diminished mitochondrial respiration and energy production capacity. Despite these reductions, the ADP/ATP ratio in BEP-control rats remained comparable to SP-control rats, indicating that ethanol exposure alone did not overtly disrupt ATP turnover at steady state (Figure 5).

Importantly, BEP rats treated with LrhGG (BEP-Lrh) showed a notable downregulation of *HIGD2A* and *AMPKα1* expression, while *MnSOD1* levels remained unchanged (Figure 2). In contrast, BEP-control and SP-control rats showed similar HIGD2A and AMPKα1 expression (Figure 2), indicating that the suppression of these mitochondrial regulators was specifically associated with *L. rhamnosus* GG treatment in the context of prior ethanol exposure. This pattern suggests that, in the heart, *L. rhamnosus* GG may induce a tissue-specific adaptive response, potentially reflecting a regulated reduction in mitochondrial activation or remodeling under recovery conditions.

Although total antioxidant capacity increased in *L. rhamnosus* GG-treated groups, this change did not correspond with restoration of NADH or ATP levels. Thus, *L. rhamnosus* GG’s cardioprotective effects appear to be limited to redox balance, with minimal impact on mitochondrial energy restoration or biogenesis in ethanol-compromised cardiac tissue. Together, these results highlight the limited and possibly regulatory effect of LrhGG on cardiac mitochondrial physiology, contrasting with its more robust benefits in the liver and bone marrow.

In the liver, mitochondrial NADH levels increased in all experimental groups (SP-Lrh, BEP-P, and BEP-Lrh) (Figure 4), suggesting elevated substrate supply or enhanced metabolic flux through the TCA cycle across conditions. However, this rise in NADH did not uniformly correspond to improved mitochondrial output. Specifically, SP-Lrh and BEP-P rats exhibited reduced ATP levels, lower ADP/ATP ratios, and diminished total antioxidant capacity (Figure 4), indicating that increased NADH alone was not sufficient to sustain energy production or redox balance in these groups.

In contrast, the BEP-Lrh group showed a coordinated recovery of mitochondrial function, including significant increases in ATP levels, ADP/ATP ratio, and total antioxidant capacity (Figure 4). This functional restoration was paralleled by upregulation of HIGD2A, MnSOD1, and AMPKα1 (Figure 2), suggesting that *L. rhamnosus* GG promotes a comprehensive adaptive response. HIGD2A upregulation likely supports respiratory supercomplex assembly, while MnSOD1 enhances antioxidant defense and AMPKα1 activation promotes energy sensing and mitochondrial biogenesis.

Together, these findings indicate that *L. rhamnosus* GG selectively enhances mitochondrial recovery in ethanol-compromised liver tissue. The probiotic appears to exert little to no benefit—and possibly a regulatory suppression—in healthy tissue (SP-Lrh) but induces mitochondrial, energetic, and antioxidant restoration in ethanol-exposed liver (BEP-Lrh). This highlights the context-dependent efficacy of *L. rhamnosus* GG, reinforcing its potential therapeutic value in conditions of metabolic stress such as alcohol-induced liver dysfunction.

The bone marrow demonstrates mild mitochondrial dysfunction following adolescent binge-like ethanol exposure. Unlike the heart and liver, which show severe and moderate damage, respectively, the bone marrow maintains relative mitochondrial stability, with indicators of metabolic stress that are effectively reversed by *L. rhamnosus* GG treatment. In the BEP-control group, ethanol exposure led to elevated NADH levels and a reduced ADP/ATP ratio, indicating a mismatch between metabolic input and ATP generation (Figure 3). This suggests subtle mitochondrial inefficiency or increased energy demand, possibly linked to immune cell activation or redox imbalance. However, mitochondrial ATP levels did not significantly drop, implying preserved overall energy production capacity.

*L. rhamnosus* GG treatment in ethanol-exposed animals (BEP-Lrh group) resulted in a restoration of the ADP/ATP ratio, a significant increase in ATP levels, and enhanced total antioxidant capacity (Figure 3), reflecting effective recovery of mitochondrial function and redox balance. This recovery was supported by increased expression of *AMPKα1*, a key regulator of mitochondrial biogenesis and energy sensing, while *MnSOD1* and *HIGD2A* levels remained relatively unchanged (Figure 2), suggesting that the antioxidant response was adequately maintained without the need for structural respiratory remodeling.

**Figure 7:**
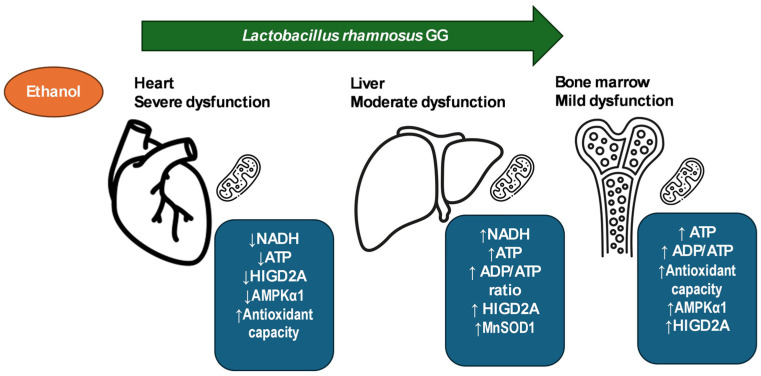
Tissue-specific effects of *Lactobacillus rhamnosus* GG on mitochondrial function in ethanol-exposed rats. This graphical model illustrates the differential impact of *L. rhamnosus* GG on mitochondrial indicators in the heart, liver, and bone marrow of rats subjected to adolescent binge-like ethanol exposure. In the heart, *L. rhamnosus* GG treatment resulted in a reduction in NADH and ATP levels and downregulation of HIGD2A and AMPKα1, with no significant change in MnSOD1, indicating limited mitochondrial recovery and redox-focused adaptation. In the liver, *L. rhamnosus* GG selectively restored mitochondrial function in ethanol-exposed rats (BEP-Lrh), as evidenced by increased ATP levels, ADP/ATP ratio, antioxidant capacity, and upregulation of HIGD2A, MnSOD1, and AMPKα1, while showing minimal benefit in non-exposed controls (SP-Lrh). In the bone marrow, LrhGG reversed ethanol-induced metabolic imbalance by increasing ATP levels, restoring the ADP/ATP ratio, and enhancing antioxidant defenses via AMPKα1 upregulation, with MnSOD1 and HIGD2A remaining stable. These findings highlight the context-dependent and tissue-specific efficacy of *L. rhamnosus* GG in restoring mitochondrial bioenergetics and redox homeostasis in metabolically distinct organs. Downward arrows (↓) indicate a decrease, and upward arrows (↑) indicate an increase in the corresponding mitochondrial or molecular parameter.

In the SP-Lrh group, minor changes were observed, indicating that *L. rhamnosus* GG had limited effect under baseline, non-stressed conditions. The bone marrow exhibits mild mitochondrial alterations in response to ethanol exposure, primarily seen as energy imbalance rather than structural or oxidative collapse. Importantly, these alterations are fully reversed by *L. rhamnosus* GG, which supports mitochondrial bioenergetics and antioxidant defenses. These findings underscore the resilience of bone marrow and its responsiveness to probiotic intervention, reinforcing its value as a marker of systemic mitochondrial adaptation in microbiota–host interaction studies. To visually summarize the tissue-specific mitochondrial responses observed in this study, a graphical model has been included to illustrate the differential effects of Lactobacillus rhamnosus GG on bioenergetic and redox parameters across the heart, liver, and bone marrow (Figure 7).

Although histological assessments were not performed in this study—limiting direct conclusions about structural tissue damage—the observed mitochondrial alterations point to distinct metabolic adaptations across tissues. While our study provides valuable insights into the tissue-specific effects of *L. rhamnosus* GG on mitochondrial function and antioxidant responses, we did not evaluate other key aspects such as mitochondrial morphology and dynamics, which are essential to fully understand the mechanisms of mitochondrial remodeling. These elements should be addressed in future studies.

Additionally, it is important to explore the specific bioactive mediators produced by *L. rhamnosus* GG—such as short-chain fatty acids (SCFAs), peptides, or exopolysaccharides—which may directly influence mitochondrial signaling and account for the observed regulation of Higd2a expression and antioxidant capacity. Although *L. rhamnosus* GG is a widely studied probiotic, the precise in vivo metabolites and pathways responsible for its mitochondrial effects remain unclear.

Future investigations should aim to determine whether the beneficial effects observed are mediated primarily by *L. rhamnosus* GG-secreted factors, by host–microbe interactions, or by a combination of both, to advance our understanding of probiotic–mitochondria crosstalk and therapeutic potential. The results obtained provide a solid foundation for future studies investigating the mechanistic pathways through which probiotics influence mitochondrial remodeling. Understanding how Higd2a and OXPHOS remodeling interact in different tissues could open new therapeutic strategies for optimizing mitochondrial health in conditions such as metabolic disorders and alcohol-induced mitochondrial dysfunction.

## 5. Conclusions

*L. rhamnosus* GG induces tissue-specific mitochondrial adaptive responses in both rats exposed to ethanol (BEP) and those exposed to saline (SP) during adolescence. The results indicate that *L. rhamnosus* GG modulates mitochondrial physiology by enhancing antioxidant defense mechanisms and activating *AMPKα1*, a key regulator of cellular energy homeostasis.

Moreover, the observed changes in Higd2a expression and the remodeling of respiratory complexes (OXPHOS) suggest that *L. rhamnosus* GG contributes to the reorganization of mitochondrial bioenergetics in response to ethanol-induced stress. Although further studies are required to fully elucidate the molecular mechanisms involved, these findings highlight the potential of probiotic interventions in mitigating mitochondrial dysfunction associated with early-life alcohol exposure.

## Figures and Tables

**Table 1:** Number of animals for each group.

Ethanol Exposure During Adolescence	Probiotics	Abbreviation Treatment in Graphics	Number of Animals
BEP	*L. rhamnosus* GG	BEP-Lrh	10
BEP	Control	BEP-P	10
SP	*L. rhamnosus* GG	SP-Lrh	10
SP	Control	SP-P	10

## Data Availability

The raw data supporting the conclusions of this article will be made available by the authors on request.

## Supplementary Materials

The following supporting information can be downloaded at: https://www.mdpi.com/article/10.3390/antiox14060627/s1, Figure S1: Quantification of *L. rhamnosus* GG (LrhGG) in feces of rats exposed to ethanol (BEP) or saline (SP) during adolescence and treated with the probiotic.

## Abbreviations

The following abbreviations are used in this manuscript:

ATP	Adenosine triphosphate
ADP	Adenosine diphosphate
NADH	Nicotinamide adenine dinucleotide reduced
Lrh	*Lactobacillus rhamnosus* GG
ROS	Reactive oxygen species
BEP	binge-like ethanol exposure
SP	saline-exposed
OXPHOS	oxidative phosphorylation
Higd2a	HIG1 Hypoxia Inducible Domain Family Member 2A
AMPKα1	AMP-Activated Protein Kinase, Catalytic, Alpha-1
MnSOD1	Manganese Superoxide Dismutase

## References

[1] Carvajal F., Lerma-Cabrera J.M., Claborn D.M. (2015). Alcohol Consumption Among Adolescents—Implications for Public Health. Topics in Public Health.

[2] Olesen M.A., Quintanilla R.A., Martin C.R., Patel V.B., Preedy V.R. (2023). Chapter 20—Alcohol consumption induces oxidative damage, neuronal injury, and synaptic impairment: Consequences for the brain health. Diet and Nutrition in Neurological Disorders.

[3] Pérez M.J., Loyola R., Canelo F., Aranguiz A., Tapia-Monsalves C., Osorio-Fuentealba C., Quintanilla R.A. (2020). NADPH oxidase contributes to oxidative damage and mitochondrial impairment induced by acute ethanol treatment in rat hippocampal neurons. Neuropharmacology.

[4] Quintanilla R.A., Pérez M.J., Aranguiz A., Tapia-Monsalves C., Mendez G. (2020). Activation of the Melanocortin-4 Receptor Prevents Oxidative Damage and Mitochondrial Dysfunction in Cultured Hippocampal Neurons Exposed to Ethanol. Neurotox. Res..

[5] Tapia-Rojas C., Torres A.K., Quintanilla R.A. (2019). Adolescence binge alcohol consumption induces hippocampal mitochondrial impairment that persists during the adulthood. Neuroscience.

[6] Tapia-Rojas C., Carvajal F.J., Mira R.G., Arce C., Lerma-Cabrera J.M., Orellana J.A., Cerpa W., Quintanilla R.A. (2018). Adolescent Binge Alcohol Exposure Affects the Brain Function Through Mitochondrial Impairment. Mol. Neurobiol..

[7] Gérard P. (2016). Gut microbiota and obesity. Cell. Mol. Life Sci..

[8] Blazewicz S.J., Barnard R.L., Daly R.A., Firestone M.K. (2013). Evaluating rRNA as an indicator of microbial activity in environmental communities: Limitations and uses. ISME J..

[9] Gheorghe C.E., Cryan J.F., Clarke G. (2022). Debugging the gut-brain axis in depression. Cell Host Microbe.

[10] Leigh S.J., Clarke G., Cryan J.F. (2022). Rewiring bugs: Diet, the gut microbiome, and nerve regeneration. Dev. Cell.

[11] Jia W., Li H., Zhao L., Nicholson J.K. (2008). Gut microbiota: A potential new territory for drug targeting. Nat. Rev. Drug Discov..

[12] Hur K.Y., Lee M.-S. (2015). Gut Microbiota and Metabolic Disorders. Diabetes Metab. J..

[13] Ji Y., Park S., Park H., Hwang E., Shin H., Pot B., Holzapfel W.H. (2018). Modulation of Active Gut Microbiota by Lactobacillus rhamnosus GG in a Diet Induced Obesity Murine Model. Front. Microbiol..

[14] Huang E., Kim S., Park H., Park S., Ji Y., Todorov S.D., Lim S.-D., Holzapfel W.H. (2021). Modulation of the Gut Microbiome and Obesity Biomarkers by Lactobacillus Plantarum KC28 in a Diet-Induced Obesity Murine Model. Probiotics Antimicrob. Proteins.

[15] Sun X., Shukla M., Wang W., Li S. (2024). Unlocking gut-liver-brain axis communication metabolites: Energy metabolism, immunity and barriers. NPJ Biofilms Microbiomes.

[16] Treven P., Mrak V., Bogovič Matijašić B., Horvat S., Rogelj I. (2015). Administration of probiotics Lactobacillus rhamnosus GG and Lactobacillus gasseri K7 during pregnancy and lactation changes mouse mesenteric lymph nodes and mammary gland microbiota. J. Dairy. Sci..

[17] Catozzi C., Cuscó A., Lecchi C., De Carlo E., Vecchio D., Martucciello A., D’Angelo L., Francino O., Sanchez Bonastre A., Ceciliani F. (2019). Impact of intramammary inoculation of inactivated Lactobacillus rhamnosus and antibiotics on the milk microbiota of water buffalo with subclinical mastitis. PLoS ONE.

[18] Markowiak-Kopeć P., Śliżewska K. (2020). The Effect of Probiotics on the Production of Short-Chain Fatty Acids by Human Intestinal Microbiome. Nutrients.

[19] Degli Esposti M., Chouaia B., Comandatore F., Crotti E., Sassera D., Lievens P.M.-J., Daffonchio D., Bandi C. (2014). Evolution of mitochondria reconstructed from the energy metabolism of living bacteria. PLoS ONE.

[20] Saint-Georges-Chaumet Y., Edeas M. (2015). Microbiota–mitochondria inter-talk: Consequence for microbiota–host interaction. Pathog. Dis..

[21] Yardeni T., Tanes C.E., Bittinger K., Mattei L.M., Schaefer P.M., Singh L.N., Wu G.D., Murdock D.G., Wallace D.C. (2019). Host mitochondria influence gut microbiome diversity: A role for ROS. Sci. Signal..

[22] Dalile B., Van Oudenhove L., Vervliet B., Verbeke K. (2019). The role of short-chain fatty acids in microbiota-gut-brain communication. Nat. Rev. Gastroenterol. Hepatol..

[23] Macfabe D.F. (2012). Short-chain fatty acid fermentation products of the gut microbiome: Implications in autism spectrum disorders. Microb. Ecol. Health Dis..

[24] McCann M.R., George De la Rosa M.V., Rosania G.R., Stringer K.A. (2021). L-Carnitine and Acylcarnitines: Mitochondrial Biomarkers for Precision Medicine. Metabolites.

[25] Lagod P.P., Naser S.A. (2023). The Role of Short-Chain Fatty Acids and Altered Microbiota Composition in Autism Spectrum Disorder: A Comprehensive Literature Review. Int. J. Mol. Sci..

[26] Silva Y.P., Bernardi A., Frozza R.L. (2020). The Role of Short-Chain Fatty Acids From Gut Microbiota in Gut-Brain Communication. Front. Endocrinol..

[27] Moos W.H., Faller D.V., Harpp D.N., Kanara I., Pernokas J., Powers W.R., Steliou K. (2016). Microbiota and Neurological Disorders: A Gut Feeling. Biores. Open Access.

[28] Obrenovich M., Jaworski H., Tadimalla T., Mistry A., Sykes L., Perry G., Bonomo R.A. (2020). The Role of the Microbiota-Gut-Brain Axis and Antibiotics in ALS and Neurodegenerative Diseases. Microorganisms.

[29] Zhang Y., Zhou L., Xia J., Dong C., Luo X. (2022). Human Microbiome and Its Medical Applications. Front. Mol. Biosci..

[30] Frye R.E., Melnyk S., Macfabe D.F. (2013). Unique acyl-carnitine profiles are potential biomarkers for acquired mitochondrial disease in autism spectrum disorder. Transl. Psychiatry.

[31] Khatri G.S., Kurian C.J., Anand A., Ka P. (2021). Gut Homeostasis; Microbial Cross Talks in Health and Disease Management. Curr. Res. Nutr. Food Sci. J..

[32] Li K., Ly K., Mehta S., Braithwaite A. (2022). Importance of crosstalk between the microbiota and the neuroimmune system for tissue homeostasis. Clin. Transl. Immunol..

[33] Comisión Nacional de Investigación Científica y Tecnológica (2009). Aspectos Bioéticos de la Experimentación Animal. 4to Taller de Bioética organizado por Comité Asesor de Bioética, FONDECYT-CONICYT.

[34] Lerma-Cabrera J.M., Arévalo-Romero C.A., Cortés-Toledo G.A., Adriasola-Carrasco A.A., Carvajal F. (2019). Emotional Reactivity to Incentive Downshift in Adult Rats Exposed to Binge-Like Ethanol Exposure During Adolescence. Front. Psychol..

[35] Carvajal F., Lerma-Cabrera J.M., Alcaraz-Iborra M., Navarro M., Thiele T.E., Cubero I. (2017). Nucleus Accumbens MC4-R Stimulation Reduces Food and Ethanol Intake in Adult Rats Regardless of Binge-Like Ethanol Exposure during Adolescence. Front. Behav. Neurosci..

[36] Ahlroos T., Tynkkynen S. (2009). Quantitative strain-specific detection of Lactobacillus rhamnosus GG in human faecal samples by real-time PCR. J. Appl. Microbiol..

[37] Doron S., Hibberd P.L., Goldin B., Thorpe C., McDermott L., Snydman D.R. (2015). Effect of Lactobacillus rhamnosus GG Administration on Vancomycin-Resistant Enterococcus Colonization in Adults with Comorbidities. Antimicrob. Agents Chemother..

[38] Ahmed H.M., Shehata H.H., El-Saeed G.S.M., Gabal H.H.A., El-Daly S.M. (2022). Ameliorative effect of Lactobacillus rhamnosus GG on acetaminophen-induced hepatotoxicity via PKC/Nrf2/PGC-1α pathway. J. Genet. Eng. Biotechnol..

[39] Shi J., Zhao G., Huang X., Li X., Ma Y., Yang K. (2023). Effects of Lactobacillus rhamnosus Supplementation on Growth Performance, Immune Function, and Antioxidant Capacity of Newborn Foals. J. Equine Vet. Sci..

[40] Vaishnavi V.V.K., Banik U., Sabesan G.S., Adhikary A.K., Parasuraman S. (2024). Evaluation of Acute and Sub-Chronic Toxicity of Lactobacillus rhamnosus GG in Sprague-Dawley Rats. Adv. Biomed. Res..

[41] Zakhari S. (2013). Alcohol metabolism and epigenetics changes. Alcohol. Res. Curr. Rev..

[42] Steiner J.L., Lang C.H. (2017). Etiology of alcoholic cardiomyopathy: Mitochondria, oxidative stress and apoptosis. Int. J. Biochem. Cell Biol..

[43] Indrio F., Salatto A. (2025). Gut Microbiota-Bone Axis. Ann. Nutr. Metab..

[44] Ludin A., Gur-Cohen S., Golan K., Kaufmann K.B., Itkin T., Medaglia C., Lu X.-J., Ledergor G., Kollet O., Lapidot T. (2014). Reactive oxygen species regulate hematopoietic stem cell self-renewal, migration and development, as well as their bone marrow microenvironment. Antioxid. Redox Signal..

[45] Schnaitman C., Greenawalt J.W. (1968). Enzymatic properties of the inner and outer membranes of rat liver mitochondria. J. Cell Biol..

[46] Trachootham D., Lu W., Ogasawara M.A., Nilsa R.D., Huang P. (2008). Redox regulation of cell survival. Antioxid. Redox Signal.

[47] Chance B. (1954). Spectrophotometry of intracellular respiratory pigments. Science.

[48] Adriasola-Carrasco A.A., Cortés-Toledo G., Arévalo-Romero C., Flores-Bastias O., Barreto M.N., Cañadas F., Cardona D., Carvajal F., Lerma-Cabrera J.M. Efecto de Lactobacillus rhamnosus (gg) Sobre Conductas de Ansiedad en Ratas Pre-Expuestas a Alcohol Durante la Adolescencia. Proceedings of the XXXVIII Congreso Anual de la Sociedad de Farmacologia de Chile, Hotel Enjoy de la Isla.

[49] Hock D.H., Reljic B., Ang C.S., Muellner-Wong L., Mountford H.S., Compton A.G., Ryan M.T., Thorburn D.R., Stroud D.A. (2020). HIGD2A is Required for Assembly of the COX3 Module of Human Mitochondrial Complex IV. Mol. Cell. Proteom. MCP.

[50] Timón-Gómez A., Garlich J., Stuart R.A., Ugalde C., Barrientos A. (2020). Distinct Roles of Mitochondrial HIGD1A and HIGD2A in Respiratory Complex and Supercomplex Biogenesis. Cell Rep..

[51] Nurrahma B.A., Tsao S.P., Wu C.H., Yeh T.H., Hsieh P.S., Panunggal B., Huang H.Y. (2021). Probiotic Supplementation Facilitates Recovery of 6-OHDA-Induced Motor Deficit via Improving Mitochondrial Function and Energy Metabolism. Front. Aging Neurosci..

[52] Ahadi N., Mahmoodzadeh Hosseini H., Halabian R., Fahimi H. (2020). Evaluation of Lactobacillus rhamnosus Antioxidant Effects on Survival of Human Mesenchymal Stem Cells. J. Appl. Biotechnol. Rep..

[53] Chi M., Jiang T., He X., Peng H., Li Y., Zhang J., Wang L., Nian Q., Ma K., Liu C. (2023). Role of Gut Microbiota and Oxidative Stress in the Progression of Transplant-Related Complications following Hematopoietic Stem Cell Transplantation. Oxidative Med. Cell. Longev..

[54] Hawrysh P.J., Gao J., Tan S., Oh A., Nodwell J., Tompkins T.A., McQuibban G.A. (2023). PRKN/parkin-mediated mitophagy is induced by the probiotics *Saccharomyces boulardii* and *Lactococcus lactis*. Autophagy.

[55] Teng Y., Wang Y., Tian Y., Chen Y.-y., Guan W.-y., Piao C.-h., Wang Y.-h. (2020). Lactobacillus plantarum LP104 ameliorates hyperlipidemia induced by AMPK pathways in C57BL/6N mice fed high-fat diet. J. Funct. Foods.

[56] Kumar H., Dhalaria R., Guleria S., Cimler R., Sharma R., Siddiqui S.A., Valko M., Nepovimova E., Dhanjal D.S., Singh R. (2023). Anti-oxidant potential of plants and probiotic spp. in alleviating oxidative stress induced by H_2_O_2_. Biomed. Pharmacother..

[57] Guo R., Scott G.I., Ren J. (2010). Involvement of AMPK in Alcohol Dehydrogenase Accentuated Myocardial Dysfunction Following Acute Ethanol Challenge in Mice. PLoS ONE.

[58] Letts J.A., Sazanov L.A. (2017). Clarifying the supercomplex: The higher-order organization of the mitochondrial electron transport chain. Nat. Struct. Mol. Biol..

[59] Salazar C., Barros M., Elorza A.A., Ruiz L.M. (2022). Dynamic Distribution of HIG2A between the Mitochondria and the Nucleus in Response to Hypoxia and Oxidative Stress. Int. J. Mol. Sci..

