# Supplementary material for: Lactobacillus rhamnosus GG Modulates Mitochondrial Function and Antioxidant Responses in an Ethanol-Exposed In Vivo Model: Evidence of HIGD2A-Dependent OXPHOS Remodeling in the Liver
Source: Antioxidants (Basel). 2025 May 23;14(6):627. doi: 10.3390/antiox14060627 (PMC12189657; doi:10.3390/antiox14060627)
Supplement: Supplementary file 1 [file antioxidants-14-00627-s001.zip › antioxidants-3590692-supplementary.pdf]

a

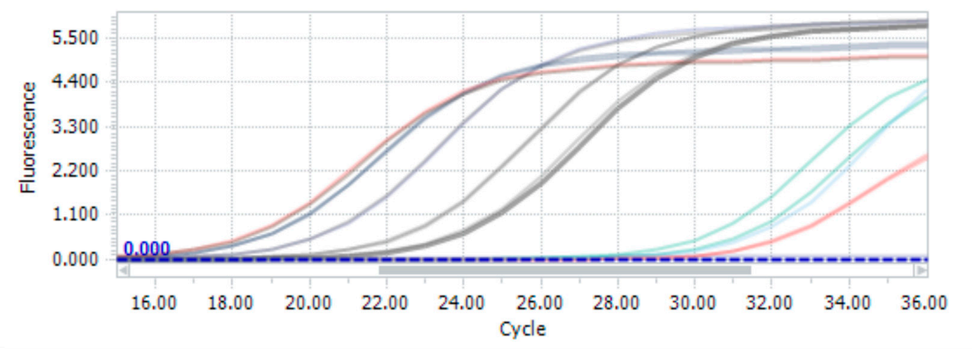

b

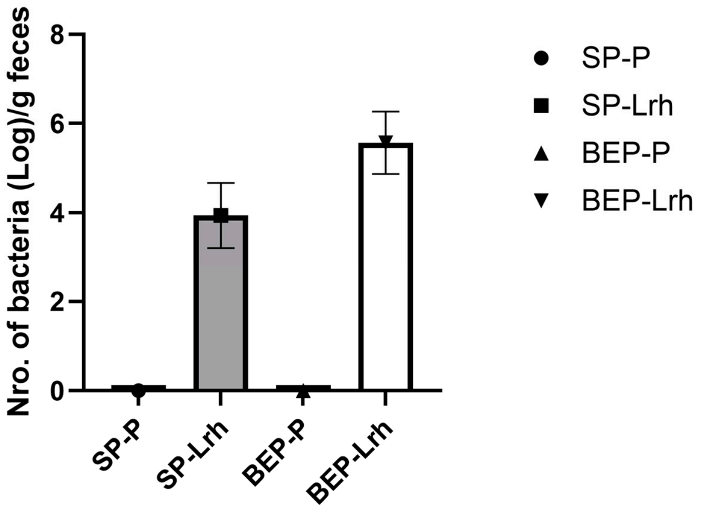

Figure S1. Quantification of *L. rhamnosus* GG (LrhGG) in feces of rats exposed to ethanol (BEP) or saline (SP) during adolescence and treated with the probiotic. (**a**) Fluorescence curve representing the standard curve used for quantification, constructed from pure cultures of *L. rhamnosus* GG and fecal samples inoculated with known concentrations of the bacterium. This standard curve was used to estimate bacterial abundance in experimental samples by qPCR. (**b**) *L. rhamnosus* GG was detected in the feces of treated rats at levels ranging from 10^5 to 10^7 CFU per gram, while it remained undetectable in the control (non-supplemented) group.
